# Biofunctional Glycol-Modified Polyethylene Terephthalate and Thermoplastic Polyurethane Implants by Extrusion-Based Additive Manufacturing for Medical 3D Maxillofacial Defect Reconstruction

**DOI:** 10.3390/polym12081751

**Published:** 2020-08-05

**Authors:** Matthias Katschnig, Juergen Wallner, Thomas Janics, Christoph Burgstaller, Wolfgang Zemann, Clemens Holzer

**Affiliations:** 1 HAGE3D GmbH, 8020 Graz, Austria; m.katschnig@hage3d.com (M.K.); office@hage3d.com (T.J.); 2 Department of Oral and Maxillofacial Surgery, University Clinic of Dental Medicine and Oral Health, Medical University of Graz, 8036 Graz, Austria; wolfgang.zemann@medunigraz.at; 3 Department of Cranio-Maxillofacial Surgery, AZ Monica and the University Hospital Antwerp, 2018 Antwerp, Belgium; 4 TCKT–Transfercenter für Kunststofftechnik GmbH, 4600 Wels, Austria; christoph.burgstaller@tckt.at; 5 Chair of Polymer Processing, Montanuniversitaet Leoben, 8700 Leoben, Austria; clemens.holzer@unileoben.ac.at

**Keywords:** biofunctional implants, glycol-modified Polyethylene terephthalate (PETG), thermoplastic polyurethane (TPU), filament-based material extrusion, patient-specific maxillofacial implants

## Abstract

This work addresses the topic of extrusion-based additive manufacturing (filament-based material extrusion) of patient-specific biofunctional maxillofacial implants. The technical approach was chosen to overcome the shortcomings of medically established fabrication processes such as a limited availability of materials or long manufacturing times. The goal of the work was a successful fabrication of basic implants for defect reconstruction. The underlying vision is the implants’ clinic-internal and operation-accompanying application. Following a literature search, a material selection was conducted. Digitally prepared three-dimensional (3D) models dealing with two representative mandible bone defects were printed based on the material selection. An ex-vivo model of the implant environment evaluated dimensional and fitting traits of the implants. Glycol-modified PET (PETG) and thermoplastic polyurethane (TPU) were finally selected. These plastics had high cell acceptance, good mechanical properties, and optimal printability. The subsequent fabrication process yielded two different implant strategies: the standard implant made of PETG with a build-up rate of approximately 10 g/h, and the biofunctional performance implant with a TPU shell and a PETG core with a build-up rate of approximately 4 g/h. The standard implant is meant to be intraoperatively applied, as the print time is below three hours even for larger skull defects. Standard implants proved to be well fitting, mechanically stable and cleanly printed. In addition, the hybrid implant showed particularly cell-friendly behavior due to the chemical constitution of the TPU shell and great impact stability because of the crack-absorbing TPU/PETG combination. This biofunctional constellation could be used in specific reconstructive patient cases and is suitable for pre-operative manufacturing based on radiological image scans of the defect. In summary, filament-based material extrusion has been identified as a suitable manufacturing method for personalized implants in the maxillofacial area. A further clinical and mechanical study is recommended.

## 1. Introduction

Repairing three-dimensional (3D) defects is a challenging part in maxillofacial surgery. Such defects can occur for many reasons, e.g., after surgical resections of pathological lesions such as cysts or tumors or after traumatic events such as traffic accidents or violent crimes. In this context, with about 40%, the lower jaw represents the highest occurrence of all facial fractures in the maxillofacial field [1]. In Germany, more than 10,000 facial mouth/jaw surgeries are operated annually, and treatment times are up to 14 h [2]. The reconstruction of maxillofacial defects is clinically important because if they are not restored, defects variable in size and form remain and might cause disfigurements and/or functional impairments. Such reconstructions always involve surgery and a patient-specific treatment plan [3].

The use of autologous bone is still the first choice in the restoration of any kind of bone defect. Autologous bone can be harvested as a vascularized or non-vascularized graft from a donor site in the same surgical procedure with the defect reconstruction. Although the autologous bone is regarded as the reconstruction gold standard for bone defects, the harvesting procedure can be time-consuming, adding additional operation time and a donor site morbidity that can cause surgery-related complications, which are exhausting for the surgeon and the patient. Furthermore, autologous bone harvesting can be impossible in medical cases where a donor site offering an adequate amount of bone is absent or simply not accessible because of general medical reasons such as bad health conditions of the patient [4]. As an alternative to autologous bone grafts, alloplastic (synthetic) materials can be used to reconstruct defects. Alloplastic materials can be formed as implants intraoperatively by the surgeon or virtually planned and customized, manufactured preoperatively according to patient-specific aspects. Commonly used alloplastic materials include metals such as titanium and titanium alloys, ceramics such as hydroxyapatite (HA) or polymers such as polyetheretherketone (PEEK) [5]. Although titanium and titanium alloys are basically biocompatible, they are relatively stiff compared to human bone. However, a bone-like modulus of elasticity is important to prevent stress shielding and/or osteolysis [6]. Additionally, metal implants are generally highly heat conducting, and thus can cause pain if patients are exposed to environmental temperature variations [7]. PEEK, on the other hand, provides low heat conduction, but also has a limited accumulation potential for cells: the surrounding bone tissue does not stay entirely healthy on implant surfaces (“halo-effect”) [8,9]. Bulk hydroxyapatite, although highly cell-friendly, is not suitable for high load bearing conditions due to its poor mechanical traits [10].

Since the discipline of maxillofacial surgery, in particular, has undergone a remarkable rate of technological innovation associated with computer assistance in the last two decades [11], patient-specific implants designed and manufactured using computer-aided design (CAD) and computer-aided manufacturing (CAM) have become a highly clinically relevant part in routine and complex individual surgical cases [12,13]. Based on a computer tomography scan (CT) or cone beam computer tomography scan (CBCT) of the patient, implants can be virtually designed using CAD software [14] and consecutively manufactured. Those services are usually clinic-externally supplied by subtractive manufacturing like milling or by additive manufacturing (AM) like powder bed fusion [7]. In contrast to AM, the cost- and material-intensive subtractive process employs milling of the 3D model from a material block in a computer numerical controlled (CNC) milling machine. Furthermore, the freedom of implant design for successful milling is limited [7].

Although the reconstruction of complex bone defects is challenging due to the unique anatomy and the variety of deficits [4,5], recent improvements in the field of CAD in combination with a compact and efficient AM could lead to precise patient-specific implants in a very short production time [11]. Thus, shorter operation times by a clinic-internal, maybe intra-operative implementation of CAD/AM would lead to less patient stress and faster healing. In addition, time-related changes in the bone structure and the extent of the lesion (bone growth) can be addressed. A material adaption to specific patient needs could also be addressed by the surgeon. For the latter demand, the extrusion-based additive manufacturing (“material extrusion” by EN ISO/ASTM 52900, edition 2017) is very promising because of the intrinsic material flexibility. At present, clinical tests of extrusion-based implants, especially internally implemented as an intra-operative option, are still in the early stages. Nevertheless, the research group MAM—Medical Additive Manufacturing, located at the University of Basel, Switzerland, has evaluated the medical approach as very promising [15,16,17]. In contrast, Vaezi et al. [18] displayed extrusion-based additive manufacturing as still not ready for clinical entry because of insufficient print results.

Hence, the goal of this study was to face the specific problems of the established maxillofacial alloplastic materials (stress shielding, heat conduction, halo-effect, mechanical performance) with an adapted material selection and to combine those materials with the extrusion-based additive manufacturing (on a filament basis) to prove the basic ability of this fabrication approach for the CAD-based reconstruction of maxillofacial defects. This study is based on the preliminary work of additive manufacturing of biofunctional implants for craniomaxillofacial surgery [7].

## 2. Materials and Methods

### 2.1. Material Selection

The implant materials were selected regarding melt flowability, mechanical toughness, mechanical stiffness, and biotoxicity. Enough flowability should allow reliable extrusion and mechanical toughness should deliver filament flexibility. Mechanical stiffness should ensure the form stability of the implant under load. The lack of biotoxicity should avoid inflammation reactions in vivo.

Optimal flowability, displayed by the melt flow rate (MFR), was determined by the evaluation of commercial filament technical data sheets [19]. The optimal MFR for material extrusion was evaluated between 5 g/10 min and 50 g/10 min. The implant requirement of the mechanical toughness and the mechanical stiffness were investigated for different polymers in previous works of Katschnig et al. [7,20]. The main results suggested a composite of a rigid and stiff polymer and a soft and tough polymer. This material hybrid delivered a synergy effect in mechanical tests, especially in the non-linear increase in the impact energy [7]. These findings can result in a stiff and at the same time tough implant. These data are given in Table 1. The bioactivity of PETG and TPU was also examined by Katschnig [7] and is given in Table 2. Moreover, the positive cell-impact of polyurethane polymers was proposed by J.A. de La Peña-Salcedo et al. [21]. Following these preliminary works, the final material choice fell on the (hard) thermoplastic polyethylene terephthalate glycol (PETG) as base polymer and the (soft) thermoplastic elastomer polyurethane (TPU) as bioactive polymer. The PETG Mimesis DP300 was supplied by Selenis (Selenis S.A, Portalegre Portugal) and the TPU Polyflex TPU95 was purchased from Polymaker (Polymaker BV, Shanghai, China).

This selection promoted the idea of using TPU outer layers (soft shell) as crack stoppers for PETG-filled (stiff core) implants in the maxillofacial area. At the same time, a TPU shell could have a cell-activating effect. The potential combination of enhanced mechanical performance and bioactive shell forms the potential biofunctionality of the fabricated implant. Extrusion-based AM opens the possibility of producing these biofunctional hybrids in one process step by dual printing.

An Acrylester–Styrol–Acrylnitril (ASA) named ApolloX Natural from Formfutura (Formfutura BV, Nijmegen, The Netherlands) was used for all printed ex-vivo bone models. The material was chosen because of the bone-like colour and good printability.

### 2.2. Filament Preparation

TPU was purchased in filament form, but PETG was only available as pellets and had to be prepared for filament-based material extrusion by filament extrusion. Payr described important influencing factors in the extrusion of high-quality filaments [22]. For a polyethylene (PE), a polypropylene (PP) and for a polycarbonate (PC) it was proven that optimal settings for high quality filaments include slow and uniform cooling of the extruded filaments. As a result, filaments could be produced in the range of 1.75 mm ± 0.05 mm in diameter, ±0.05 mm in ovality and without vacuoles. Slow and uniform filament cooling can be done with symmetrical air cooling and a slow draw-off speed. This is achieved, for example, by the table-top 3devo Advanced 1.0 filament extruder (3devo B.V., Utrecht, The Netherlands), which was therefore used for the extrusion of PETG filaments. A further advantage was the easy processing of small raw material batches below 5 kg. The 3devo Advanced 1.0 is equipped with an optical filament diameter measurement, thus has an automated quality control during production. Table 3 lists the machine specifications.

### 2.3. Clinical Data Preparation

The anatomical defect localizations were chosen to be clinically relevant and comparable to the surgical routine, e.g., after the surgical resection of tumors and pathologic bone lesions or after expanded traumatic injuries including heavy bone loss.

#### 2.3.1. Maxillofacial Defect Data Source

A physiologically preserved anonymous human cadaver mandible, donated within the “anatomical body donation program” of the Institute for Macroscopic and Clinical Anatomy at the Medical University of Graz, was used to create two comprehensible bone defects in the lower chaw.

A first bone defect (implant A) was created in the anatomical area of the right mandibular angle (approx. 3.0 cm × 2.0 cm × 1.0 cm), including the right oblique line, cortical bone and the infra-alveolar nerve, as it often occurs after the resection of neoplastic processes that infiltrate the bone. The aim was to digitally scan the defect and the previously resected defect positive. The aim was to achieve the subsequent surgical filling of the defect with a printed replication of the defect positive.

A second bone defect (implant B) was created by the resection of the muscular process (pterygoid process) on the left side of the human mandible (approx. 3.5 cm × 3.0 cm × 1.0 cm), as a potential result of a pathological bone lesion resection or of a congenital missing or malformed anatomical hard tissue structure. The aim was to replicate the second defect, i.e., to “model” the defect positive digitally and then to print the implant.

All defect preparations were done with a commercially available oscillating bone saw and a blade width of 0.8 mm, using water cooling to secure further structures in the mandible cadaver bone.

Regarding the localization of the reconstruction sites for implant A and B, the mandible was chosen for both defect creations because it provided highly complex geometry for additive manufactured implants, a bone to be subject to naturally occurring strong biomedical forces and clinical highly relevant fracture sites [1,2,3].

#### 2.3.2. Imaging of Anatomical Structures

All defects were scanned using the CT scanner Siemens Sensation 64 (Siemens Medical Solutions, Malvern, PA, USA). The scanners are regularly subjected to quality control evaluations; scanning procedures were done with a standard scanning protocol. High-resolution images with an in-plane resolution of 0.98 × 0.98 mm^2^ of the craniomaxillofacial area and a slice thickness of 1 mm were provided by the Department of Oral and Maxillofacial Surgery (Medical University of Graz, Graz, Austria) in digital imaging and communications in medicine(DICOM) file format.

#### 2.3.3. Segmentation and STL Model Creation

All defects were converted and segmented with the help of Invesalius v3.1 (Campinas, Brazil) a free medical open source software used to generate virtual reconstructions of structures in the human body [23]. The segmentation was done by bi-level thresholding. The additional modeling of implant B was done with Meshmixer 3.5 (Autodesk Inc., San Rafael, CA, USA).

#### 2.3.4. Post-Processing of the STL Models

Any post-processing of the STL models was performed using 3D Builder (Microsoft Corporation, Redmond, WA, USA) and Meshmixer 3.5 (Autodesk Inc., San Rafael, CA, USA).

### 2.4. Material Extrusion Machines

For all single extrusion prints, the cartesian industrial printer HAGE3D medmex (HAGE3D GmbH, Graz, Austria) was used. All dual extrusion prints were carried out with the cartesian industrial printer HAGE3D 84L-A (HAGE3D GmbH, Austria). Table 4 shows the machine specifications.

The medmex machine used an air-cooled direct drive printhead (abbr. SDK) with synchronized profile wheels and mosquito hotends (Slice Engineering, Gainesville, FL, USA). The 84L-A machine used a water-cooled high friction feeding system printhead (abbr. HFFS) with shortened aluminium hot ends for precise melt control.

### 2.5. Slicing and Print Parameters

All implants and ex-vivo bone models were sliced using the software Simplify3D Version 4.0 (Simplify3D Inc., Cincinnati, OH, USA) with a brass nozzle of 0.4 mm diameter using the settings summarized in Table 5, Table 6 and Table 7. Those settings were based on a previous process development by the author [7]. The heatable print bed was covered by polyetherimide (PEI) foil. For dual printing, an additional value setting and a tool change script were required and are shown in Table 8 and Table 9.

After finalizing the printing job, the printed parts were detached from the cooled down print bed with a spatula and stored under standardized conditions for more than 72 h before subsequent tests were conducted.

### 2.6. Clinical Evaluation

Defect reconstructions were done ex-vivo with conventional surgical methods as they are also routinely used intraoperatively. The fixation of each implant was done with commercially available osteosynthesis microplates and screws (MedArtis AG, Basel, Switzerland). These plates and screws are also used intraoperatively in the clinical routine in facial reconstructive and bone fixation procedures and can generally remain in the patient without the need of second operation for their removal.

## 3. Results

### 3.1. Implant Models

**Figure 1:**
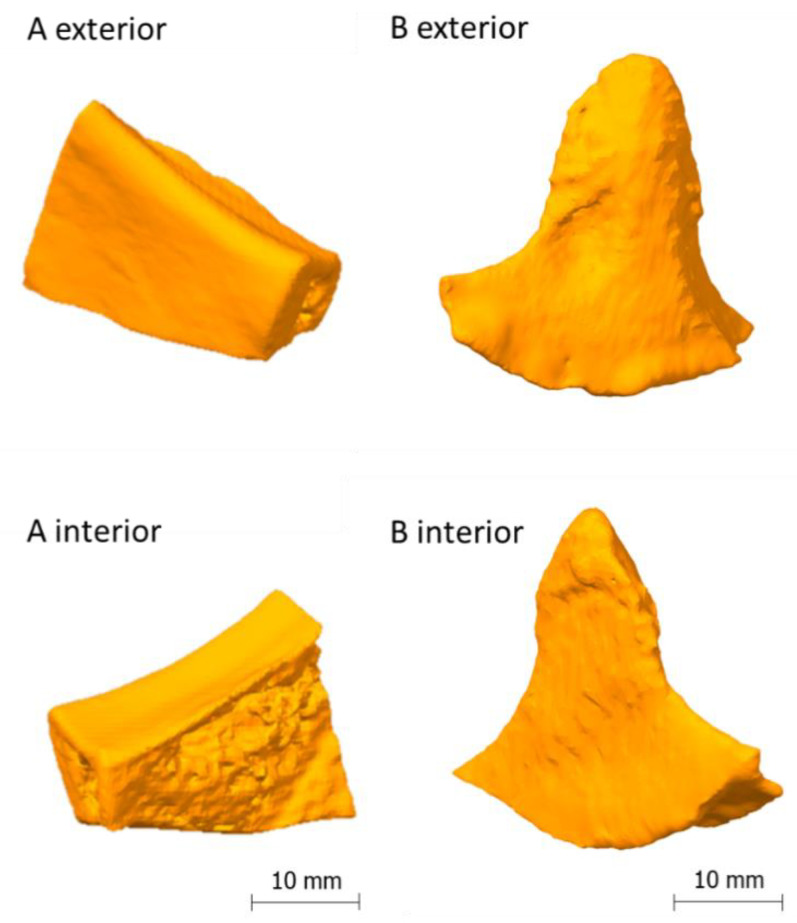
On the left side: model of implant A [7], on the right side: model of implant B.

Figure 1 shows the final STL models of implant A and implant B, which were obtained through imaging, segmentation and STL modeling.

### 3.2. Slicing

A slicing strategy was created for a standard implant made of PETG and a performance implant made of PETG (core) and TPU (shell). The standard implant should be manufactured by single printing and the performance implant by dual printing.

**Figure 2:**
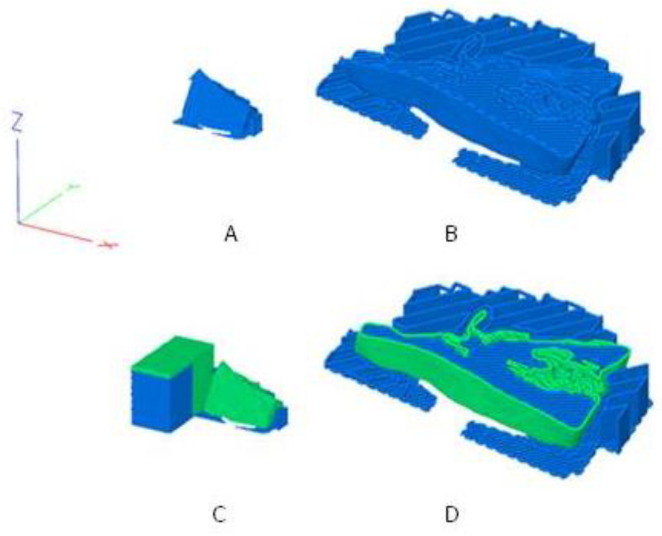
The sliced and orientated maxillofacial implant A [7]; (**A**): standard implant; (**B**): standard implant cut at Z = 5 mm and enlarged; (**C**): performance implant with TPU shell (green) and PETG core (blue); (**D**): performance implant cut at Z = 5 mm and enlarged; additionally shown in 3: the prime tower to the left of the implant.

An upright orientation in the build room was chosen to minimize the support structures and at the same time sufficiently stabilize the implant during printing. In addition, care was taken to ensure that the layer orientations correspond as far as possible to the principal stress directions in a biaxial stress state of the implant. For dual printing, a primetower was also printed. The tower ensures that the nozzle is filled after the tool change. Figure 2 shows the sliced implants including orientation in the build room, layer orientation and the material sections of PETG and TPU.

Print times calculated by Simplify3D are given in Table 10. For dual printing, the primetower is included in the print time.

### 3.3. Implant Printing

**Figure 3:**
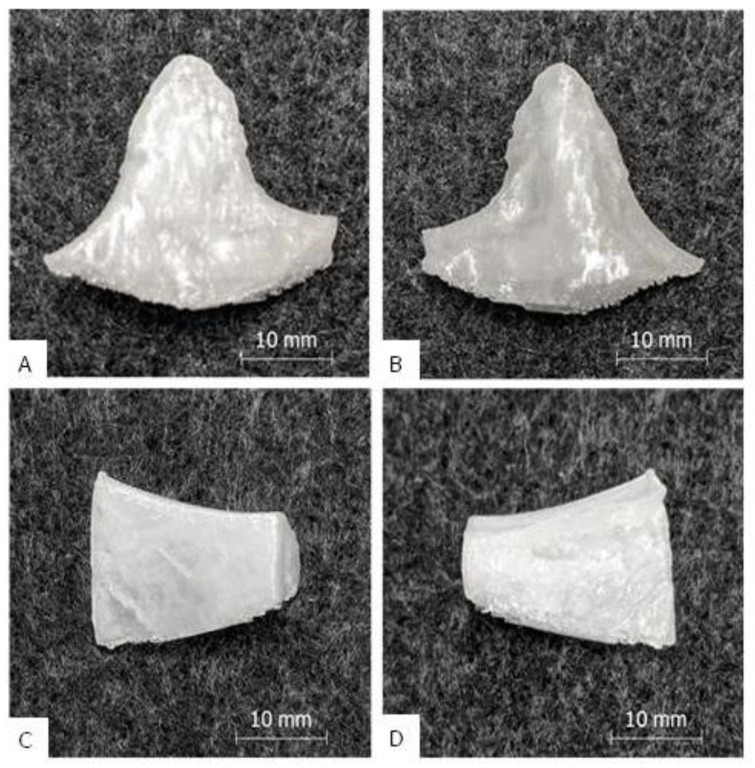
Results of the single print: Picture (**A**) shows the outside of implant B, picture (**B**) the inside. Picture (**C**) [7] shows the outside of implant A, picture (**D**) [7] the inside (with porous inner bone structure).

The PETG single print of implant A and B yielded good results, which are shown in Figure 3A,B. After the manual removal of the support structure, which came off easily and without residue, the implants could be used without further post processing. The surfaces of the implants were generally clean. Very rough structures were visible on the interior of implant A, which were caused by the natural trabecular bone structure of the model, and on the bottom of implant B, which were caused by the support interfaces. The appearance of the implants was reminiscent of milk glass with a silvery and shiny shimmer. There were no cracks or extrusion inhomogeneities and only slight layer start marks were visible. The implants appeared firm and stiff and a manual compression and bending test did not reveal any typical “crackling” indicating underextrusion and/or a lack of interlayer adhesion. The haptic surface quality was generally hard and smooth. The printing time for implant A was 27 min and for implant B 30 min, which was at maximum 1% longer than the predicted printing time. The build-up rate was approximately 10 g/h.

**Figure 4:**
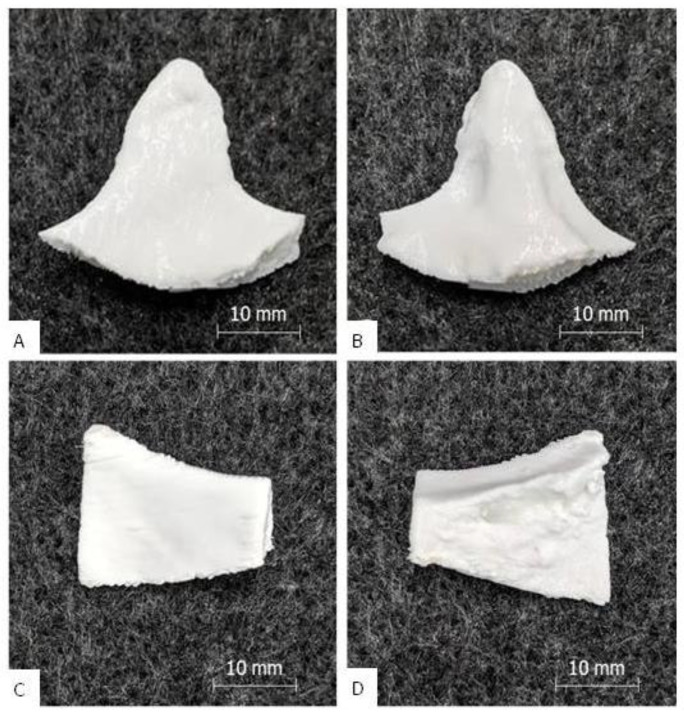
Results of the dual print: picture (**A**) shows the outside of implant B, picture (**B**) the inside; picture (**C**) [7] shows the outside of implant A, picture (**D**) [7] the inside (with porous inner bone structure).

The PETG/TPU dual print of implant A and B exhibited good results, which are shown in Figure 4. Removal of the PETG support structure from the TPU outer layer was more difficult than with the standard implants. The supported interfaces had to be carefully ablated with key files to obtain an acceptable clean surface. In contrast, the non-supported surface of all implants was smooth and clean and the PETG core material was completely enclosed by the TPU shell. On the interior side of implant A, rough structures were visible due to the cancellous bone structure. The appearance of the implants was pure white and shiny and was determined by the TPU. There were no cracks or extrusion inhomogeneities and only slight layer start marks. The implants felt strong and rigid, and a manual compression and bending test also showed no “crackling”. Only the peripheral corners were stiffer than the main body, as only TPU shell layers without PETG core material were printed here due to the small wall thickness. The general haptic surface texture was softer and less smooth compared to the PETG prints. The printing time for implant A was 2 h and 50 min and, for implant B, 2 h and 59 min, which was at maximum 11% longer than the predicted printing time. The build-up rate was approximately 4 g/h.

**Figure 5:**
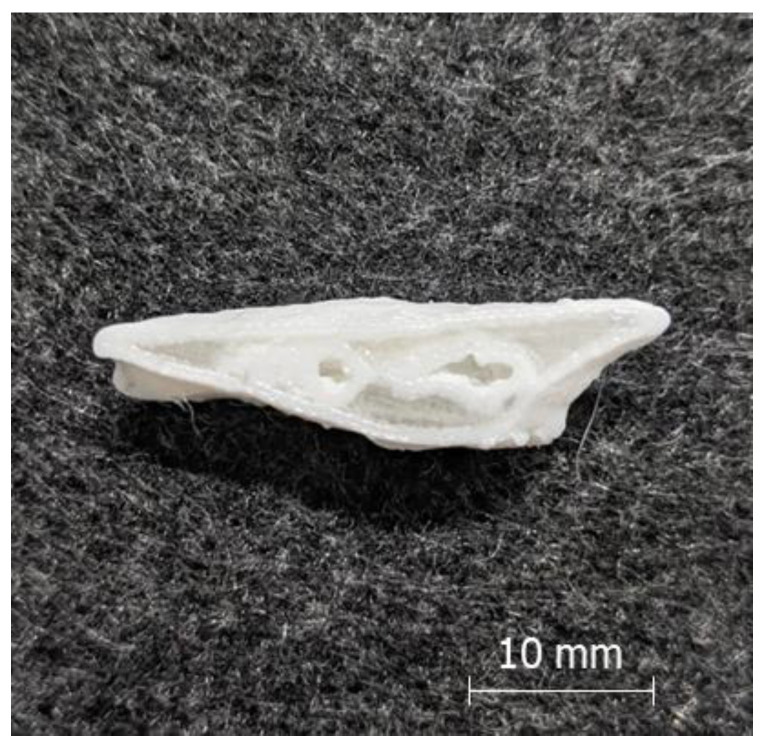
Structure of a printed performance implant (implant B, top view, cut at Z = 8 mm); white: TPU shell, transparent: PETG core.

Figure 5 shows the hybrid structure of a performance implant. Printing was stopped before completion at Z = 8 mm to obtain a sectional top view.

### 3.4. Clinical Suitability for Use

Under the aspect of clinical suitability, only the accuracy of fit (implant adaption to the defect) and the assembly capability (implant fixation in the defect) are addressed in this work. An evaluation of both the mechanical implant behavior ex-vivo/in-vivo and the real implant use during an operation were not part of this work.

**Figure 6:**
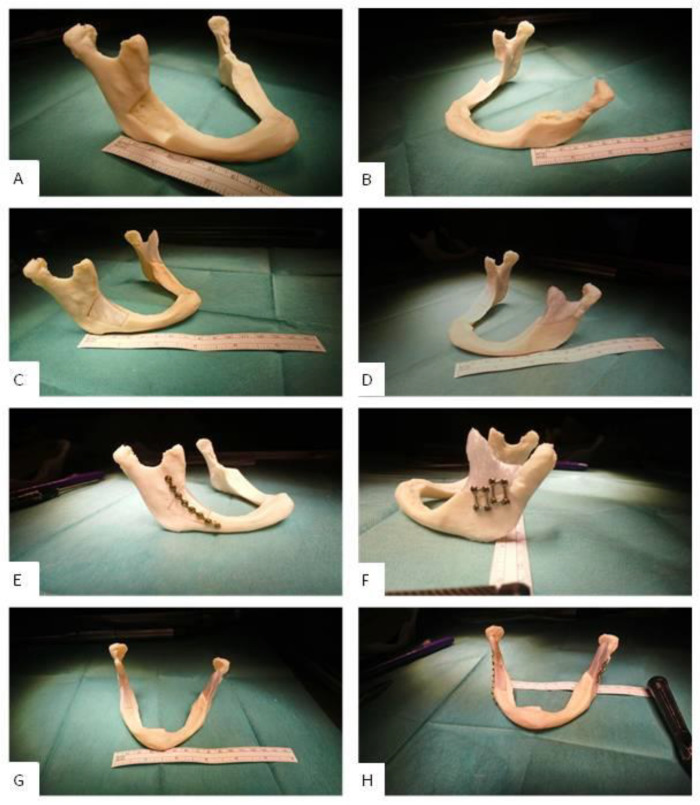
Clinic-internal assembly of the maxillofacial standard implants A [7] and B with a mandibular mini plate system from Medartis AG. The self-tapping screws were 5 to 7 mm long. (**A**): lesion A (lateral); (**B**): lesion B (lateral); (**C**): inserted implant A (lateral); (**D**): inserted implant B (lateral); (**E**): mounted implant A (lateral); (**F**): mounted implant B (lateral); (**G**): inserted implants A and B (frontal); (**H**): mounted implants A and B (frontal).

For simplification, only the standard implants were manually embedded in an associated bone model and checked for easy insertion, smooth transitions to the bone, good adaptation to the skull surface contour and the correct distance to the bone margins. The assembly capability was defined by the screwability with surgical fixatives and the holding power of the fixation. Figure 6 shows the results.

The fit of implant A and B was particularly good (cp. Figure 6C,D). The countering of the jaw (mandibular countering) was anatomically correct and aesthetically satisfying. After fixation, the implant reconstructed the defect satisfactorily. The transition to the bone was without visible height differences; the distances to the bone margins were in the ideal range of approximately 1 mm to 2 mm. The assembly could be carried out without any occurring problems; the screw connection did not produce any cracks or splinters. The screws were tight, and the implants were sufficiently fixed to the bone without remaining movements.

## 4. Discussion

The main task in this work was to search for alternatives to the established alloplastic implant materials with an adapted material selection and combine those new materials with the extrusion-based additive manufacturing on a filament basis to produce ready-to-use reconstructions of maxillofacial defects. From a manufacturing perspective, this can be affirmed based on the achieved results.

The (STL-) models “implant A” and “implant B”, made by semi-automatic segmentation and defect reconstruction with open source software, delivered a precise adaptation to the region of implantation. The three-dimensional bone contour was well reconstructed. The models had no mesh defects and the slicing was performed smoothly. The model quality was comparable to already presented CAD-based mandible reconstructions [24,25].

A clean and complete extrusion process could be carried out for both the standard and performance implants. The optimized printing parameters derived from [7] could be confirmed. In dual printing, the adhesion between the TPU shell and the PETG core seems to be good. The surfaces are homogeneous and have a process-specific resolution (±0.1 mm [26]). The exceptions were slightly frayed areas on the supported surfaces of the performance implants, which had to be smoothed in post-processing. The reason for the surface damage was probably the good adhesion between the PETG support and the TPU shell, if the defined offsets of 0.3 mm are bypassed by extrusion errors. TPU is known for its increased adhesiveness [27], so if there is any offset bridging of extruded material, the contact adhesion tends to be strong. In addition, slight traces of overheating in the TPU shell were found. The reason for this may have been a local overheating by tool path-enforced low layer times (<10 s) [28].

**Figure 7:**
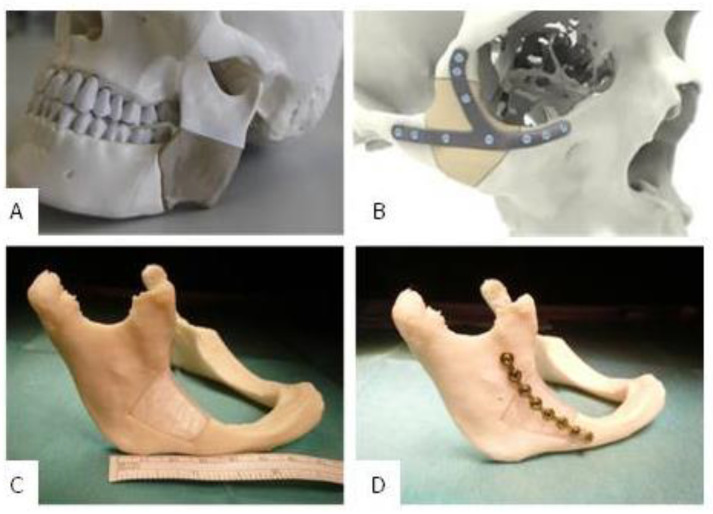
(**A**): Fitted and additively manufactured polyetheretherketone (PEEK) implant by GBN Systems GmbH/Kumovis GmbH [2]; (**B**): fitted and additively manufactured PEEK implant by KLS Martin Group [29], (**C**): fitted PETG implant A [7]; (**D**): fitted and mounted PETG implant A [7].

The assembly ability of implant A and B was clinically proven in a printed ex-vivo bone model. Figure 7 shows a qualitative comparison between an already published result of fitted and mounted PEEK implants that are conventionally available for a clinical use and a fitted and mounted PETG implant A. One can see that the clinical fixation with osteosynthesis microplates and screws possibly does not harm the implants and leads to aesthetically good results. Furthermore, the implant fits to the three-dimensional bone contour.

Every new AM-based patient-specific implant (PSI) fabrication effort must compete with the already established (and often commercialized) technologies and materials. Looking at extrusion-based AM with thermoplastics, the benchmark material is PEEK. PEEK PSIs are used clinically in a wide medical field [30]. Various studies conducted with PEEK in the reconstruction of maxillofacial defects have shown good postoperative aesthetic and functional results without any complications [31,32,33,34].

**Figure 8:**
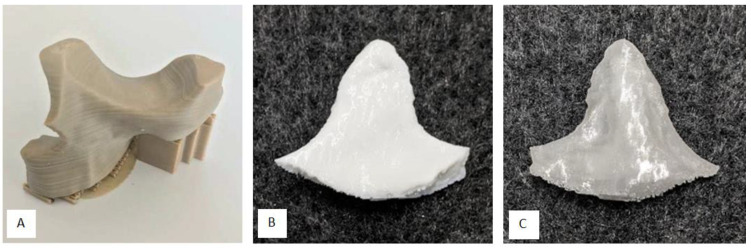
(**A**): Lightweight midface-zygomatic bone patient-specific implant (PSI) with support structures for immediate replacement [30]; (**B**): muscular process (left side of the mandible, exterior) PSI (performance implant B); (**C**): muscular process (left side of the mandible, interior) PSI (standard implant B).

However, there are also reports of PEEK post-operative complications and implant failures [35,36], although with better performance compared to titanium meshes [36], for example, and the difficulty to print PEEK [37]. From a clinical point of view PEEK PSIs may cause local infection and increased soft tissue scarring due to a limited biocompatible surface, especially in anatomical areas where strong muscles are crossing and moving strong bones, for example in the lower jaw. Furthermore, PEEK is not ideal for dual printing due its high extrusion temperature and consecutively beneficial material composites like hard–soft-combinations, for example, rib replacements and fixtures are not easy to achieve. Moreover, producing compounds with PEEK and bioactive fillers like HA is difficult [7]. Those disadvantages should motivate researchers to establish other thermoplastics and thermoplastic elastomers as alternatives. Figure 8 depicts a visual comparison between a maxillofacial PEEK implant and a standard/performance implant B. With the results of this publication and preliminary works of the authors in mind, PETG or TPU as mono material implant or PETG/TPU as material composite are potentially a promising substitute for PEEK.

However, there are some drawbacks in using PETG and TPU instead of PEEK, such as the non-eligibility for autoclave sterilization (ultraviolet radiation sterilization or ethylene oxide sterilization could be alternatives) or the minor load-bearing capability, which must be addressed in further studies. Maxillofacial reconstructions need high-strength implants, especially if there is not enough supporting biological bone structure. This is especially true in the mandible, where the highest forces of the whole maxillofacial complex can occur that are punctually at a maximum average of 700 N in a healthy human. These high-strength forces result from bite forces that influence the bone. However, clinically, for as long as 6 weeks after an operation, bite forces are typically between 0 N and 100 N for incisal edge front loading and between 0 N and 200 N for molar edge rear loading [38,39]. Thus, those value ranges represent clinically relevant load limits for the mechanical testing of implants in the maxillofacial complex [40]. After 6 weeks, the bone healing is known to be biologically stable enough and thus, together with the implant, a biological stability can be achieved [41,42]. Another important aspect that needs to be investigated further is the technology-specific mechanical anisotropy in printed parts. This means the part is normally strongest in the direction of the extruding tool path and relatively weaker in the two remaining part axes, vertically to the extruding tool path (layer bonding regions). This anisotropy should be addressed in the slicing strategy considering the in-vivo implant orientation

## 5. Conclusions and Future Work

The presented technical approach proved to be sufficiently fast, clean, and precise to exactly reconstruct maxillofacial structures ex-vivo. If an intraoperative fabrication is considered or if non-risk patients are clinically involved, a fast in-house printed PETG standard implant could potentially serve as an alternative for maxillofacial bone reconstruction. For patients who are either at risk of implant rejection or high impact stress, the TPU/PETG performance implant is a potential implant solution. It has a TPU shell that is biofunctional and crack-stopping and a PETG core that gives strength. The impact strength is synergistically increased; the risk of fracture and splintering is low. The disadvantage of performance implants is the longer manufacturing time. In summary, standard implants could make second operations obsolete by manufacturing them during surgery and performance implants could compensate for the disadvantages of currently used implant materials such as casual rejection reactions (titanium alloys etc.) or fracture susceptibility (HA etc.).

Concerning the study design, a pre-clinical ex-vivo defect creation and evaluation was done because new fabrication strategies and material combinations (PETG/TPU) were used. An in-vivo study within a clinical setting is planned as a following future work project. However, to reduce this ex-vivo limitation in our study design, we used a physiologically preserved high quality human cadaver (Institute for Macroscopic and Clinical Anatomy, Medical University of Graz), which completely consists of human cellular tissue and naturally simulates a clinical setting regarding geometry and mechanical behavior. The study design of this work was chosen according to previously successfully performed investigations of new methods for a clinical use that suggest a first pre-clinical assessment when new materials or technologies should be introduced as routine procedures in maxillofacial surgery [1,40,43].

In general, the new requirements of the standards ISO 13485:2016 (introduction to quality management for medical devices), VDI 3405 (additive manufacturing processes) and ISO 5832 (implant certification) will pose questions in particular for the additive manufacturing of implants, which need to be answered. The focus of those requirements is on the clinical evaluation of medical devices, post-market surveillance systems and quality management for in-house production in hospitals. The current standards also attach great importance to questions of approval and liability for printed implants [44]. The central question here will be whether the risk of implant failure due to manufacturing errors can be internalized, i.e., ultimately borne by the medical facility and its employees or whether the risk will continue to be externalized by external service manufacturing. A possible way out would be the separation into emergency medical operations, which require the time savings and flexibility of in-house manufacturing, and standard procedures, which make standardized external manufacturing appear reasonable. Extrusion-based AM, more precisely filament-based material extrusion, will support the flexible, fast and low-cost ready-to-use in-house manufacturing with a compact and clean printing process and the possibility of patient-specific material design. The authors therefore recommend a systematic evaluation of the complete benefit chain from data generating to clinical study of the presented technology including quality documentation and risk management. A successful result would be a relevant and vital step towards the clinical acceptance and potential use of filament-based material extrusion for patient-specific implants in maxillofacial defect reconstruction.

## Figures and Tables

**Table 1:** Mechanical characteristics [7].

Technical Parameters	Unit	PETG	TPU	PETG/TPU
Burst force (instrumented puncture test)	N	551 ± 55	2325 ± 631	1048 ± 131
Puncture energy (instrumented puncture test)	J	3.9 ± 0.6	19.5 ± 2.0	41 ± 9.8

**Table 2:** Medical characteristics [7].

Medical Parameter	Unit	PETG	TPU
Cell viability (CellTiter-Glo, reference: 54.284 ± 4915)	RLU	74.805 ± 3242	92.598 ± 7700

**Table 3:** 3devo filament extruder specifications [7].

Screw Configuration	Three-Zone, Shearing Elements, Hardened
Maximum extrusion temperature	225 °C
Nozzle diameter	3 mm
(Pulled-off) filament diameter	1.75 mm ± 0.05 mm
Resolution of diameter measurement	±0.05 mm
Machine control software	3devo version 1.1.2

**Table 4:** Performance specifications of the printers.

Parameter	Medmex	84L-A
Print bed	glass/PEI	glass/PEI
Print area (X × Y)	250 mm × 210 mm	600 mm × 400 mm
Print height (Z)	200 mm	350 mm
Extrusion temperature	285 °C (max)	450 °C (max)
Print bed temperature	110 °C (max)	130 °C (max)
Build room temperature	RT (20 °C–30 °C)	85 °C (max)
Print (machine) resolution in X-Y	0.1 mm (<0.1 mm)	0.1 mm (0.05 mm)
Print (machine) resolution in Z	0.1 mm (<0.1 mm)	0.1 mm (0.05 mm)
Print speed	200 mm/s (max)	100 mm/s (max)
Machine control system	Prusa firmware 3.5.0	Sigmatek control

**Table 5:** Glycol-modified Polyethylene terephthalate (PETG) parameter table, nozzle: 0.4 mm [7].

Parameter	Unit	Medmex	84L-A
Print bed temperature	°C	90	90
Extrusion temperature	°C	230	230
Extrusion multiplier	/	0.88	0.50
Layer height	mm	0.18	0.18
Extrusion width	mm	0.48	0.48
Shrinkage compensation (X-Y-Z)	%	+0.3	+0.3
Cooling intensity	%	100	100
Retraction length	mm	2	2
Rapid motion speed	mm/s	200	200
Printing speed	mm/s	50	50
Wiping	mm	0	5
Angle bridging infill	°	45	45
Bridging speed multiplier	/	0.9	1
Bridging extrusion multiplier	/	1.1	1
Support density	%	30	30
Support angle	°	45	45
Support offset horizontal	mm	0.3	0.3
Support offset vertical	mm	0.3	0.3

**Table 6:** Thermoplastic polyurethane (TPU) parameter table, nozzle: 0.4 mm [7].

Parameter	Unit	Medmex	84L-A
Print bed temperature	°C	60	60
Extrusion temperature	°C	240	245
Extrusion multiplier	/	0.95	0.52
Shrinkage compensation (X-Y-Z)	%	0	0
Cooling intensity	%	100	100
Retraction length	mm	2	2
Rapid motion speed	mm/s	200	200
Printing speed	mm/s	50	50
Wiping	mm	0	5
Angle bridging infill	°	45	45
Bridging speed multiplier	/	1	1
Bridging extrusion multiplier	/	1	1
Support density	%	10	10
Support angle	°	45	45
Support offset horizontal	mm	0.3	0.3
Support offset vertical	mm	0.3	0.3

**Table 7:** Acrylester–Styrol–Acrylnitril (ASA) parameter table, nozzle: 0.4 mm [7].

Parameter	Unit	Value
Print bed temperature	°C	85
Extrusion temperature	°C	245
Extrusion multiplier	/	0.95
Shrinkage compensation (X-Y-Z)	%	0.47
Cooling intensity	%	50
Retraction length	mm	1
Rapid motion speed	mm/s	200
Printing speed	mm/s	60
Wiping	mm	5

**Table 8:** Dual printing parameters [7].

Parameter	Unit	Value
Tool 0	/	TPU
Tool 1	/	PETG
Support	/	PETG
Retraction length on tool change	mm	6
Retraction speed on tool change	mm/s	20
Primetower location	/	south-west to home position
Primetower width	mm	25
Number of contours (TPU)	/	2
Contour extrusion width (TPU)	mm	0.96
Angle core layer/shell layer	°	45

**Table 9:** Tool change script [7].

G1 X0 F15000; move to waste management
G1 Y30 F15000; move to waste management
G92 E0; set E0 to zero
T[new_tool]; change tool
M6
T[new_tool]
{IF NEWTOOL=0} M104 S200 T1; idle right extruder
{IF NEWTOOL=0} M104 S[extruder0_temperature] T0; heat left extruder
{IF NEWTOOL=0} M109 S[extruder0_temperature] T0; wait for left extruder
{IF NEWTOOL=1} M104 S200 T0; idle left extruder
{IF NEWTOOL=1} M104 S[extruder1_temperature] T1; heat right extruder
{IF NEWTOOL=1} M109 S[extruder1_temperature] T1; wait for right extruder
G1 E10 F180; extrude 10 mm filament
G92 E0; set E0 to zero
G1 X60 F15000; move 60 mm forward

**Table 10:** Calculated print times.

Implant	Calculated Print Time
Implant A Standard	25 min
Implant A Performance	2 h 30 min
Implant B Standard	29 min
Implant B Performance	2 h 38 min
